# Implications of Microbiota and Immune System in Development and Progression of Metabolic Dysfunction-Associated Steatotic Liver Disease

**DOI:** 10.3390/nu16111668

**Published:** 2024-05-29

**Authors:** Jelena Popov, Tijana Despot, David Avelar Rodriguez, Irfan Khan, Eugene Mech, Mahrukh Khan, Milan Bojadzija, Nikhil Pai

**Affiliations:** 1 Boston Combined Residency Program, Boston Children’s Hospital & Boston Medical Center, Boston, MA 02115, USA; jelena.popov@childrens.harvard.edu; 2 College of Medicine and Health, University College Cork, T12 YN60 Cork, Ireland; 121115766@umail.ucc.ie (T.D.); ikhanmacalumni@gmail.com (I.K.); 3 Department of Pediatric Gastroenterology, Hepatology & Nutrition, The Hospital for Sick Children, University of Toronto, Toronto, ON M5G 1E8, Canada; david.avelarrodriguez@sickkids.ca; 4 School of Medicine, University College Dublin, D04 C1P1 Dublin, Ireland; eugene.a.f.mech@gmail.com; 5 Department of Pediatrics, Faculty of Health Sciences, McMaster University, Hamilton, ON L8S 4L8, Canada; khanm285@mcmaster.ca; 6 Department of Medical Sciences, Faculty of Health Sciences, McMaster University, Hamilton, ON L8S 4L8, Canada; 7 Department of Internal Medicine, Subotica General Hospital, 24000 Subotica, Serbia; mbojadzija@yahoo.com; 8 Division of Gastroenterology, Hepatology and Nutrition, McMaster Children’s Hospital, Hamilton, ON L8S 4L8, Canada; 9 Department of Pediatrics, Perelman School of Medicine, University of Pennsylvania, Philadelphia, PA 19104, USA; 10 Division of Gastroenterology, Hepatology, and Nutrition, Children’s Hospital of Philadelphia, Philadelphia, PA 19104, USA

**Keywords:** microbiome, immunology, metabolism, metabolic dysfunction-associated steatotic liver disease, metabolic dysfunction-associated steatohepatitis

## Abstract

Metabolic dysfunction-associated steatotic liver disease (MASLD) is the most prevalent type of liver disease worldwide. The exact pathophysiology behind MASLD remains unclear; however, it is thought that a combination of factors or “hits” act as precipitants for disease onset and progression. Abundant evidence supports the roles of diet, genes, metabolic dysregulation, and the intestinal microbiome in influencing the accumulation of lipids in hepatocytes and subsequent progression to inflammation and fibrosis. Currently, there is no cure for MASLD, but lifestyle changes have been the prevailing cornerstones of management. Research is now focusing on the intestinal microbiome as a potential therapeutic target for MASLD, with the spotlight shifting to probiotics, antibiotics, and fecal microbiota transplantation. In this review, we provide an overview of how intestinal microbiota interact with the immune system to contribute to the pathogenesis of MASLD and metabolic dysfunction-associated steatohepatitis (MASH). We also summarize key microbial taxa implicated in the disease and discuss evidence supporting microbial-targeted therapies in its management.

## 1. Introduction

Metabolic dysfunction-associated fatty liver disease (MASLD) is a metabolic disorder characterized by hepatic steatosis > 5% in the setting of cardiometabolic risk factors. An estimated 25–30% of patients progress to the most severe state of the disease, known as metabolic dysfunction-associated steatohepatitis (MASH), which is characterized by inflammation, hepatocyte apoptosis, and the development of liver fibrosis [1]. Further progression of disease can ultimately lead to cirrhosis and hepatocellular carcinoma. MASLD is commonly comorbid with obesity, type 2 diabetes (T2D), and metabolic syndrome (MetS) [2], with a cumulative global prevalence of 25% as of 2020 [3]. Common complications of MASLD include cardiovascular disease, hepatocellular carcinoma, colorectal cancer, non-hepatic malignancies, T2D, and chronic kidney disease [4]. The complications of MASH are similar to those of MASLD, including hepatocellular carcinoma, liver failure, and cirrhosis [5]. Current estimates indicate that MASLD will surpass all other indications for liver transplants by the year 2030 [6].

Historically, MASLD was referred to as nonalcoholic fatty liver disease (NAFLD) to differentiate its etiology from alcohol-associated fatty liver disease, given the similarities in histopathologic findings of both disorders [7]. More recently, there has been increased interest in modifying this exclusion-based nomenclature to better capture the influence of metabolic dysfunction in driving disease pathogenesis, provide further clarity on diagnostic criteria, and reduce stigma associated with the disease. In 2023, an international panel of content experts, clinicians, and patient advocate groups arrived at a consensus decision to rename the disease MASLD. The diagnosis of MASLD requires the presence of at least one of five cardiometabolic risk factors, which were selected based on previously validated markers associated with metabolic syndrome and cardiovascular disease. The cut-offs for the risk factors vary slightly by source but may be defined as (1) BMI ≥ 23 kg/m^2^ (or alternatively ≥ 25 kg/m^2^); (2) fasting serum glucose ≥ 100 mg/dL (≥5.6 mmol/L), diagnosis of type 2 diabetes, or treatment for type 2 diabetes; (3) blood pressure ≥ 130/85 mmHg or on an antihypertensive drug treatment; (4) plasma triglycerides ≥ 150 mg/dL (≥1.70 mmol/L) or on a lipid-lowering treatment; and (5) plasma high-density lipoprotein (HDL) cholesterol ≤ 40 mg/dL (≤1.0 mmol/L) for males and ≤50 mg/dL (≤1.3 mmol/L) for females, or on a lipid-lowering agent. Despite MASLD being both a semantic and a diagnostic criterion shift, it is important to note that previously published NAFLD research is still applicable to MASLD. Based on an evaluation of patients in the LITMUS (Liver Investigation: Testing Marker Utility in Steatohepatitis) consortium, it was found that 98% of NAFLD patients satisfied the MASLD diagnostic criteria, thus allowing for all previously published work regarding pathogenesis, biomarkers, and clinical trials to remain applicable [8].

## 2. Pathophysiology

The pathophysiology of MASLD is complex and multifactorial. It was initially explained by the “two-hit” hypothesis, which proposed that the first “hit” involves steatosis [9] stimulated by factors such as sedentary lifestyle, metabolic syndrome, and insulin insensitivity. The second “hit” involves the formation of reactive oxygen species (ROS), which subsequently catalyze lipid peroxidation, hepatocyte apoptosis, steatohepatitis, and fibrosis. This hypothesis explains why steatosis does not always progress to steatohepatitis and why second “hits”, such as alcohol or particular drugs, may be required for disease progression. Over time, this hypothesis has evolved into the “multiple parallel hits” hypothesis, which proposes that several factors are involved in the development of MASLD, including diet (particularly a Western diet high in refined sugars and animal fats and low in fiber) [10], genetic and epigenetic factors, and metabolic factors including insulin resistance and disordered lipid metabolism [11], as well as alterations in the gut microbiota [12]. Thus, a multitude of factors, including environmental, biochemical, and epigenetic factors, contribute to the pathogenesis of MASLD.

**Figure 1:**
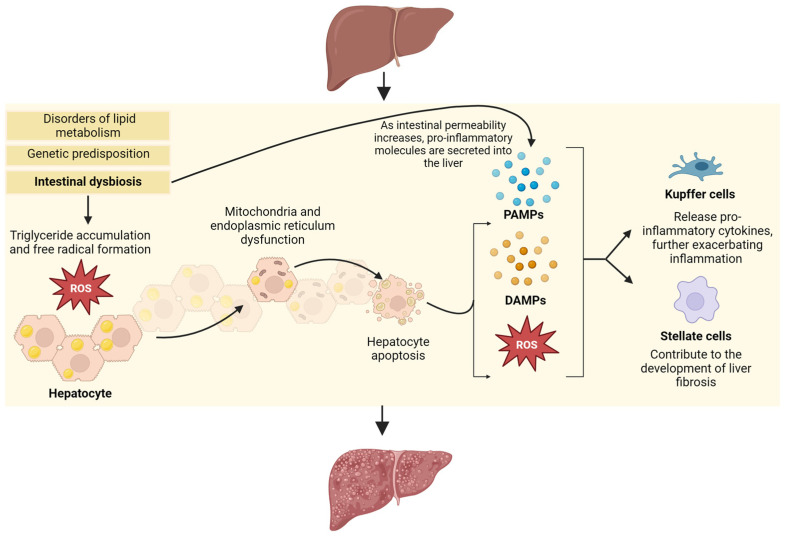
The pathophysiology of MASLD/MASH. A combination of genetic, metabolic, and microbial factors alters the ratio of anti-inflammatory-to-pro-inflammatory signaling pathways, which impairs intestinal mobility and barrier function. This imbalance promotes the gradual accumulation of triglycerides in hepatocytes, increased oxidative stress, the dysregulation of intracellular machinery, and subsequent hepatocyte apoptosis. Inflammation is exacerbated by the ongoing generation of reactive oxygen species (ROS), as well as pathogen-associated molecular patterns (PAMPs) and damage-associated molecular patterns (DAMPs), which leak out from the impaired intestinal barrier. This pro-inflammatory environment culminates in the activation of hepatic Kupffer cells and stellate cells, leading to ongoing inflammation and fibrosis.

A combination of the aforementioned hypotheses suggests that genetic predisposition, disorders of lipid metabolism, and intestinal dysbiosis shift the balance from anti-inflammatory to pro-inflammatory signaling. This may impair gut motility and intestinal barrier function [13], thereby promoting the gradual accumulation of triglycerides in hepatocytes and the formation of free radicals. This oxidative stress subsequently causes mitochondrial and endoplasmic reticulum dysfunction, resulting in hepatocyte apoptosis. Further inflammation may be driven by the formation of additional ROS and exposure to gut-derived pathogen-associated molecular patterns (PAMPs) and damage-associated molecular patterns (DAMPs) as the intestinal tract becomes increasingly leaky. Finally, these stimulate the activation of both Kupffer cells, which further release pro-inflammatory cytokines, and stellate cells, which contribute to liver fibrosis (Figure 1) [13].

While the exact pathogenic mechanisms underpinning MASLD remain elusive, a growing body of evidence is supporting the role of multiple factors working in concert to drive disease development and progression. This narrative review will focus on the intersection between the intestinal microbiome and the immune system in driving hepatocyte inflammation and progression to MASLD. It will also discuss current evidence supporting the role of emerging microbiota-based therapies in managing the disease.

## 3. Immunological Implications

The hepatic microenvironment represents a diverse site of immunological activity comprising both the innate and adaptive branches of the immune system. Long-lived resident immune cells include Kupffer cells, CD8+ resident memory T cells (TRM), and ILC1s [14]. Circulating immune cells include natural killer cells (NK), γδ T cells, CD4+ and CD8+ αβ T cells, monocytes, B cells, invariant natural killer T cells (iNKT), dendritic cells (DCs), and mucosal-associated invariant T cells (MAIT). During disease development and subsequent progression of MASLD to MASH, there is a significant remodeling of the hepatic immune environment, which ultimately promotes hepatocellular injury and subsequent fibrosis through the upregulation of pro-inflammatory pathways [14].

### 3.1. Innate Immune Response

The liver microenvironment contains numerous innate immune cells that display altered prevalence and function in liver diseases, including MASLD/MASH [14]. These innate immune cells include DCs, neutrophils, and macrophages. DCs play a vital role in the induction of the adaptive immune response through the presentation of antigens to naïve T cells in lymph nodes [15]. Both types of conventional DCs, i.e., CD103+ cDC1s and CD11b+ cDC2s, accumulate in the murine hepatic MASH environment; however, their role in the pathogenesis of disease is currently unknown [15,16,17]. In patients with MASH, there is an increase in hepatic cDC1 cells; however, the exact role and prevalence of cDC2 cells has similarly not yet been elucidated [17]. Overall, further research into the mechanisms and contribution of DC-influenced hepatic injury in the context of MASLD/MASH is required.

Another prominent innate immune cell type contributing to the pathophysiology of MASLD/MASH are neutrophils. Neutrophils are innate immune cells that are typically involved in acute infectious processes, but they can also contribute to chronic inflammatory disorders through ROS, cytokine secretion, and neutrophil extracellular traps (NETs) [18]. It has been shown that neutrophil numbers are increased in both murine models of MASH and MASH patients [18,19,20,21]. As the depletion of neutrophils in murine models of MASH only yielded improvements in the early stages of disease, it has been hypothesized that neutrophils are mediators in early-stage MASH progression [20]. Also, the disruption of NETs in murine MASH models limits hepatic inflammation, injury, and fibrosis [19,21].

Another prominent innate immune mediator in MASLD/MASH is represented by phagocytic macrophages. The hepatic cellular microenvironment contains both liver-resident macrophages, called Kupffer cells, and circulating monocyte-derived macrophages [22]. Murine models of MASH have indicated increased numbers of macrophages in the liver, with decreased numbers of tissue-resident Kupffer cells [23]. It has been shown that the murine tissue-resident Kupffer cells in MASH models have an increased inflammatory transcription profile and lower expression of the surface marker TIMD4, indicating that they may have arisen from circulating monocytes [23]. These TIMD4-low Kupffer cells derived from circulating monocytes demonstrate decreased storage of hepatic triglycerides but an increased ability to cause hepatic injury [24]. Another prominent macrophage population in the liver are the inflammatory macrophages derived from Ly6C+ circulating monocytes [25]. A previous study demonstrated that murine hepatic monocyte-derived macrophages showed similar characteristics to desmin+ HSC populations, which may indicate involvement in hepatic fibrosis [26,27]. This finding was also present in human MASH patients [26]. Furthermore, several studies showed that a pro-inflammatory TREM2+ CD9+ monocyte-derived macrophage population was present in human MASH patients [27,28]. Overall, innate immune cell populations including macrophages, neutrophils, and dendritic cells play a role in the pathogenesis of MASLD/MASH. Further investigation is required to determine their exact role in disease progression and onset.

### 3.2. Adaptive Immune Response

During disease progression to MASH, there is a significant increase in hepatic B-cell and T-cell populations, leading to augmented inflammation, injury, and fibrosis, which may influence disease course [14]. B cells belong to the adaptive branch of the immune system and produce immunoglobulins, present antigens, and secrete numerous cytokines [29,30,31]. In the context of MASH, CD19+ B cells are pro-inflammatory and influence inflammation and injury via both B-cell receptor-mediated immune responses and MyD88-dependent innate immune mechanisms [32]. This is most likely because of increased gut permeability and intestinal inflammatory mediators. While it has been shown that intrahepatic B cells produce less pro-inflammatory IL-6 and TNFα cytokines compared with macrophages and neutrophils, there is a correlation between increased hepatic lobar inflammation and fibrosis and increased B-cell numbers in MASH patients [32,33]. In particular, an antigen-specific, high-affinity, antibody-producing lineage of B cells called B2 cells are associated with MASH progression [33]. The implications of B cells in MASH have also been demonstrated by the correlation between increased serum levels of immunoglobulin A (IgA) and advanced hepatic fibrosis in both mice and humans [34]. B-cell depletion in mice is also associated with reduced hepatic fibrosis [33].

Furthermore, T cells are a vital component of the adaptive immune response and can be phenotypically characterized into CD4+ helper T cells (Th) and CD8+ cytotoxic T cells [14,35]. CD4+ helper T cells can be characterized into Th1, Th2, and Th17 subsets according to differential cytokine production of IFNγ, IL-4 and/or IL-13, and IL-17, respectively [14]. CD8+ T cells produce IFNγ, TNF, and cytotoxic molecules including perforins and granzymes [14]. With respect to Th1 cells, it has been shown that MASH mouse models deficient in the production of IFNγ demonstrate decreased hepatic injury and fibrosis [36]. Also, CXCL10 is an IFNγ-inducible chemokine important for cellular chemotaxis. CXCL10 expression is increased in MASH patients, and the depletion of CXCL10 in murine MASH models has been documented to decrease steatosis, liver injury, and fibrosis [37]. While Th1 cells have been implicated in the pathogenesis of MASLD/MASH in both murine models and patients, it is important to consider that production of pro-inflammatory IFNγ is not exclusive to Th1 cells and that the interplay of T cells subsets in MASH must be further studied. Furthermore, while the role of Th2 cells in MASH is unclear, it has been documented that serum levels of IL-13 and hepatic IL-13R expression are increased in MASH patients [38]. Th17 cells play a role in both homeostasis related to intestinal barrier integrity and inflammatory responses in the context of pathogens [14]. It has been shown that hepatic Th17 numbers and associated genes are increased in MASH patients [39,40]. Th17 prevalence is also increased in murine models of MASH, particularly the CXCR3+ subset, which may play a role in the pathogenesis of disease [41,42,43]. The depletion of the Th17 cytokine IL-17A was also shown to be protective in the context of wild type mice fed a MASH-inducing diet, whereas the administration of recombinant IL-17A led to augmented hepatic damage, steatosis, injury, and fibrosis [42,43]. Overall, CD4+ helper T cells play a role in the pathogenesis of MASH, as demonstrated by the depletion of CD4+ cells in a humanized mouse model of MASH leading to hepatic fibrosis [44].

Cytotoxic CD8+ T cells represent a separate branch of T-cell subsets involved in the adaptive immune response. Hepatic CD8+ T-cell numbers, particularly CD8+ T cells expressing CXCR6, are increased in both murine models and patients of MASH [34,45]. CXCR6+ CD8+ T cells in murine models of MASH have been shown to upregulate the expression of the exhaustion marker PD1, and subsequent CD8+ T-cell activation via PD-1 blockade exacerbated MASH development [46]. Furthermore, perforin-1-deficient mice had increased numbers and activation of hepatic CD8+ T cells, which accelerated MASH development [47].

Overall, the interplay among immune cells belonging to both the innate and adaptive immune systems plays an important role in the development of MASLD/MASH. Recent studies have also demonstrated that other hematological and immune components, including platelets and innate-like T-cell populations such as iNKT cells and MAIT cells, may also be involved in the pathogenesis of disease [14]. Ultimately, further investigation is required to elucidate the interactions among immune cells in the context of MASLD/MASH progression.

## 4. The Role of the Microbiome

The intestinal tract is home to approximately 1013–1014 microorganisms [48], which serve as important sources of hormones, metabolites, and immunomodulators for the gut and other extra-intestinal organs. The liver is an extra-intestinal organ which shares an intimate bidirectional functional relationship with the intestinal tract. It receives approximately 70–80% of its blood supply from the portal vein, which drains the intestines, and is thus exposed to the nutrients and pathogens of the gut. Emerging evidence is uncovering the important role of the gut microbiota in the development of MASLD and disease progression to MASH.

### 4.1. Compositional Changes of the Gut Microbiome

While consensus is currently lacking on what constitutes a MASLD-specific gut microbiome, several studies point to key changes in the microbial composition of affected individuals compared with controls. A recent meta-analysis [49] that included 15 studies found a gut microbial signature in patients with MASLD characterized by increased abundance of Proteobacteria, *Escherichia*, *Prevotella*, and *Streptococcus*, and decreased levels of *Coprococcus*, *Faecalibacterium*, and *Ruminococcus* compared with healthy controls [50,51]. Similar microbial patterns have been found in individuals with MASH, in whom increased levels of Proteobacteria and *Escherichia* were identified [52]. In a recent study [53], two microbiota subpopulations and clinical phenotypes were identified in patients with MASLD, where one resembled the healthy controls’ gut microbiome (increased *Enterobacteriaceae*) and was associated with body fat percentage and serum levels of LPS, whereas the other was characterized by an imbalanced gut microbiome (increased *Prevotella* species) and was associated with elevated levels of serum IL-6. A prospective study found that patients with MASLD exhibited decreased levels of *Methanobrevibacter* and increased levels of *Dorea formicigenerans*, a species that is also increased in individuals with obesity [54]. Table 1 summaries key gut microbiota alterations and metabolic pathways in adult and pediatric populations affected by MASLD and MASH.

**Table 1:** Alterations in microbial compositions and metabolic pathways.

HUMAN ADULT STUDIES				
Author	Population	Samples	Main Effects on Microbiota	Main Effects on Metabolites
Adams et al., 2020 [55]	58 MASLD (minimal–mild fibrosis), 9 MASLD (severe fibrosis), and 55 HCs	Fecal and serum samples	Severe (F3-F4) liver fibrosis: - ↑ Firmicutes, Proteobacteria, and Actinobacteria. - ↑ *Actinomycetaceae* and *Lachnospiraceae.* - ↓ Bacteroidetes. - ↓ *Bacteroidaceae* and an unclassifiable *Bacteroidales* family.	- ↑ primary and secondary conjugated bile acids and secondary unconjugated bile acids in severe fibrosis group. - ↑ CDCA and GCA in both MASLD groups. - ↑ DCA, GCA, GCDCA, and GDCA in MASLD severe fibrosis group.
Chen et al., 2020 [56]	538 MASLD (lean and obese) and 30 lean HCs	Fecal and serum samples	Lean versus obese MASLD patients: - ↑ *Erysipelotrichaceae* UCG-003, *Clostridiales* order (*Ruminococcus*, *Clostridium sensu stricto* 1, *Romboutsia*, and *Ruminococcaceae* UCG-008). - ↓ *Ruminiclostridium* and *Streptococcus*. - Lean NAFLD compared with lean HCs: - ↑ *Dorea*; ↓ *Marvinbryantia* and *Christensellenaceae* R7 group.	- ↑ total primary and secondary conjugated bile acids in lean MASLD compared with lean HCs. - ↑ total, primary, and secondary bile acids in severe fibrosis group. - ↓ DCA, CDCA, and glycochenodeoxycholic acid in lean versus obese MASLD. - ↑ GCA in lean versus obese MASLD. - ↑ DCA and UDCA in mild fibrosis (F0-F1) lean versus obese groups. - ↓ FGF19 in more advanced fibrosis. - ↑ FGF19 in lean versus obese MASLD. - ↓ C4 in lean versus obese MASLD.
Delik et al., 2022 [50]	20 MASLD and 20 HCs	Intestinal biopsies	↔ Firmicutes ↑ Gram-negative bacteria ↑ Phylum Proteobacteria ↓ microbial diversity ↓ *Bacteroidetes* and *Actinobacteria*	N/A
Demir et al., 2022 [57]	78 obese and non-obese MASLD (40 fibrosis F0-F1 and 38 fibrosis F2-F4), 54 NASH, and 16 HCs	Fecal and serum samples	- Order Pleosparales and order Saccharomycetales, *Candida argentea*, and *Penicillium* sp. were associated with F0-F1 fibrosis. - *Mucor* sp., *Cyberlindnera* *jadinii*, *C*. *albicans*, and *Saturnispora* sp. *Babjeviella inositovora* were associated with F2-F4 fibrosis. - *Babjeviella inositovora/S. cerevisiae, Mucor* sp.*/S. Cerevisiae*, and *Hanseniaspora/S. Cerevisiae* were associated with MASH. - ↑ *C. albicans* plasma IgG in MASLD with advanced fibrosis.	- *C. albicans*/*S. cerevisiae* was associated with ↑ ALT and AST and lower HDL. - *Mucor* sp./*S. cerevisiae* was associated with ↑ serum glucose and AST.
Hullar et al., 2021 [58]	511 MASLD and 1033 HCs	Fecal and blood samples	Pooled ethnicities MASLD compared with HCs: - ↓ Shannon diversity index. - ↑ Fusobacteria, Bacteroidetes, and Proteobacteria.	Pooled ethnicities and genders, MASLD compared with HCs: - ↑ secondary bile acid metabolism and carbohydrate metabolism.
	400 Japanese American individuals (178 MASLD and 222 HCs)		Japanese American individuals: - ↑ Family *Lachnospiraceae (Blautia, Shuttleworthia, Ruminococcus gnavus group*, and *Eubacterium eligens group*) and Family *Enterobacteriaceae* (*Enterobacter*). - ↓ Families *Lachnospiraceae (Coprococcus 2, Lachnospiraceae UCG-003, Lachnospiraceae NK4A136, Bacteroides pectinophilus group*, and *Coprococcus 2*), Ruminococcaceae (*UBA1819, DTU089, Ruminococcaceae UCG-014, Ruminiclostridium 6, CAG-352*, and *Ruminococcaceae UCG-005*), *Prevotellaceae (Prevotella 1, Prevotella 2*, and *Paraprevotella*), and *Erysipelotrichaceae* (*Erysipelatoclostridium* and *Catenibacterium*).	- ↑ carbohydrate metabolism in males.
	257 African American individuals (38 MASLD and 219 HCs)		African American individuals: - ↑ Families *Enterobacteriaceae* (*Escherichia-Shigella* and *Enterobacter*), *Lachnospiraceae* (*Tyzzerella*), and *Lactobacillaceae* (*Lactobacillus*). - ↓ Families *Prevotellaceae* (*Prevotella 7* and *Prevotella 2*), Fusobacteriaceae (Fusobacterium), *Veillonellaceae* (*Megamonas*), *Lachnospiraceae (Lachnospira, Lachnospiraceae UCG-003, Bacteroides pectinophilus group, Lachnospiraceae NK4A136, Eubacterium eligens group, Anaerostipes*, and *Coprococcus 1*), Marinifilaceae (*Odoribacter*), *Clostridiaceae* (*Clostridium sensu stricto 1*), *Pasteurellaceae* (*Haemophilus*), *Bacteroidales* RF16 group uncultured bacteria, *Ruminococcaceae* (*Eubacterium coprostanoligenes group*), *Synergistaceae* (*Cloacibacillus*), and *Christensenellaceae* (*Christensenellaceae R-7 group*).	
	316 White individuals (67 MASLD and 249 HCs)		White individuals: - ↑ Families *Ruminococcaceae* (*Flavonifractor, UBA1819*, and *Negativibacillus*), *Enterobacteriaceae* (*Klebsiella, Escherichia-Shigella*, and *Enterobacter*), *Lachnospiraceae (Ruminococcus gnavus, Eisenbergiella*, and *Tyzzerella*), *Coriobacteriaceae* (*Collinsella*), *Fusobacteriaceae* (*Fusobacterium*), *Prevotellaceae* (*Alloprevotella*), and *Coriobacteriaceae* (*Collinsella*). - ↓ Families *Puniceicoccaceae* uncultured, *Victivallaceae* (*Victivallis*), *Barnesiellaceae* (*Coprobacter*), *Lachnospiraceae* (*Coprococcus-2, Lachnospira, Lachnospiraceae ND3007 group, Butyrivibrio, Eubacterium oxidoreducens group, Coprococcus 2, *and* Lachnospiraceae AC2044 group*), *Ruminococcaceae* (*Ruminococcus 1*).	
	325 Latino individuals (143 MASLD and 182 HCs)		Latino individuals: - ↑ Families *Lachnospiraceae (Ruminococcus gnavus*,* Lachnoclostridium*, and *Lachnospiraceae UCG-008*) and *Pasteurellaceae* (*Aggregatibacter*). - ↓ Families *Prevotellaceae* (*Alloprevotella*), *Ruminococcaceae* (*Ruminococcaceae NK4A4214 group *and* Ruminococcaceae UCG-005*), and *Erysipelotrichaceae* (*Catenibacterium*).	- ↓ ethanol production in females.
	246 Native Hawaiian individuals (85 MASLD and 161 HCs)		Native Hawaiian individuals: - ↑ Families *Veillonellaceae (Megamonas), Burkholderiaceae *(*Parasuterella*)*, Veillonellaceae (Megamonas), and Lactobacillaceae *(*Lactobacillus*)* *. - ↓ Families *Odopmarinaceae* (*Idiomarina*), *Enterobacteriaceae* (*Klebsiella*), *Erysipelotrichaceae* (*Erysipelatoclostridium*), *Ruminococcaceae* (*Negativibacillus*, *Intestinimonas*, and *Ruminococcaceae UCG-005*), *Clostridiaceae* (*Clostridium sensu stricto 1*), and *Lachnospiraceae* (*Bacteroides pectinophilus group*).	- ↑ butyrate, glycine, serine, threonine, taurine, hypotaurine, branched chain amino acids, and fatty acid metabolism in females. - ↑ nitrous oxide reductase in males.
Jee et al., 2022 [53]	16 MASLD (microbiome similar to (P_HC-like_) and dissimilar (P) from health controls)	Fecal and serum samples	- ↑ *Enterobacteriaceae* in P_HC-like_ patients. - ↑ *Prevotella* in P patients.	- ↑ dicarboxylic acid sugar degradation in P_HC-like_. - ↑ heme b biosynthesis and sulfate reduction in P patients.
Jiao et al., 2021 [59]	86 MASLD and 38 HCs	Fecal and serum samples	- ↓ bacterial diversity. - ↑ Clostridia class (*Eubacterium siraeum*, *Clostridium bolteae*, and *E. coli*). - ↑ Bacteroidia class (*B. ovatus* and *Bacteroides stercoris*). - ↑ *Eubacterium siraeum*, *Clostridium bolteae*, and *E. coli*. - ↓ Bacteroidia class (*Bacteroides dorei* and *Alistipes shahii*). - ↓ Clostridia class (*Eubacterium eligens*, *Eubacterium hallii*, and *Faecalibacterium prausnitzii*).	- ↑ secondary bile acid biosynthesis, benzoate degradation, biosynthesis of ansamycins, and oxidative phosphorylation. - ↑ 7α-hydroxysteroid dehydrogenase, 3α-hydroxysteroid dehydrogenase, and bile acid-coenzyme A ligase
Lang et al., 2020 [60]	73 MASLD (37 with F0–F1 fibrosis, 36 with F2–F4 fibrosis, 29 with NAS 0–4, and 44 with NAS 5-8/LCI) and 22 HCs (9 without liver disease and 13 with mild primary biliary cholangitis)	Fecal and serum samples	More advanced disease: - ↓ intestinal viral diversity and bacteriophages. - ↑ *Escherichia*, *Enterobacteria*, and *Lactobacillus* phages. - ↓ *Lactococcus* and *Leuconostoc* phages.	N/A
Leung et al., 2022 [54]	90 MASLD and 90 HCs	Fecal and serum samples	- ↔ α-diversity, Shannon index, and Simpson’s index at family, genus, and species levels. - ↓ *Methanobrevibacter.* - ↑ *Slackia*, *Dorea formicigenerans.*	- ↓ geranylgeranyl diphosphate biosynthesis (hydroxymethyglutaryl–coenzyme A (CoA) reductase). - ↓ mevalonate pathway (mevalonate kinase). - ↑ phosphatidate metabolism. - ↑ CA degradation. - ↑ 8,11,14-eicosatrienoic acid (linked with obesity). - ↓ Phenyllactic acid (linked with reduced reactive oxygen species).
Mouzaki et al., 2016 [61]	12 MASL, 16 MASH, and 25 HCs	Fecal and serum samples	↓ Bacteroidetes in MASH. ↓ *Clostridium leptum* in MASH.	↑ CA in MASLD and MASH ↑ CDCA in MASH ↓ LCA in MASLD and MASH ↑ Primary-to-Secondary BA Ratio in MASH ↑ Total BAs in MASH
Oh et al., 2021 [62]	22 MASLD and 44 HCs	Fecal samples	MAFLD compared with HCs: - ↓ *Eubacterium, Faecalibacterium, Ruminococcus, *and* Oscilibacter*. - ↑ *Enterococcus, Megamonas, *and* Veillonella*. - ↔ *Bacteroidetes*. - ↑ *Proteobacteria* (*Enterobacter, Escherichia, *and* Citrobacter*). - ↓ Butyrate-producing bacteria (*Anaerostipes, Coprococcus, Eubacterium, Roseburia, Faecalibacterium, Odoribacter, Oscillibacter, Subdoligranulum, Butyricimonas, Alistipes, Pseudoflavonifractor, Clostridium, Butyricicoccus, *and* Flavonifractor*). - ↑ Alcohol-producing bacteria (*Klebsiella* and *Escherichia*).	N/A
Sui et al., 2021 [63]	59 NHS non-diabetic patients and 32 HCs	Fecal samples	NHS compared with HCs: - ↑ Phyla Proteobacteria and Fusobacteria. - ↑ Families *Enterobacteriaceae*, *Coriobacteriaceae*, *Fusobacteriaceae*, *Moraxellaceae*, *Actinomycetaceae*, and *Carnobacteriaceae.* - ↓ Family *Dehalobacteriaceae.* - ↑ Genera *Shigella*, *Collinsella*, *Megamonas*, *Leuconostoc*, *Acinetobacter*, and *Actinomyces.* - ↓ Genera *Lachnospira*, *Anaerostipes*, *Butyricimonas*, *Odoribacter*, *Anaerofustis*, and *Dehalobacterium*.	- ↑ kynurenine, 3-indoleacetonitrile, tryptamine, 3-(3-indolyl)-2-oxopropanoic acid, L-phenylalanine, L-homophenylalanine, 3-(2-Hydroxyphenyl)propanoic acid, CDCA, and CA. - ↓ L-tryptophan and acetate.
Wang et al., 2016 [64]	43 MASLD and 83 HCs	Fecal and serum samples	MASLD compared with HCs: - ↓ Shannon diversity index. - ↑ Simpson’s diversity index. - ↓ Firmicutes (particular class Clostridia and families *Lachnospiraceae*, *Ruminococcaceae*, *Lactobacillaceae*, and *Peptostreptococcaceae*). - ↑ Bacteroidetes (Bacteroidia). - ↑ Gram-negative populations.	- ↓ short-chain fatty acids-producing and 7α-dehydroxylating bacteria. - ALT and uric acid negatively correlate with Firmicutes and positively correlate with Bacteroidetes. - γ-GT negatively correlates with Firmicutes. - Increased plasma ALT associated with depleted genera (*Pseudobutyrivibrio*, *Moryella*, *Anaerosporobacter*, *Roseburia*, *Ruminococcus*, *Anaerotruncus*, and *Lactobacillus*).
Wang et al., 2021 [65]	505 MASLD (306 mild, 174 moderate, and 25 severe disease) and 1393 HCs	Breath test and serum samples	- ↑ *H. pylori* infection in females with NAFLD (no significance in males).	N/A
Yun et al., 2019 [66]	76 MASLD and 192 HCs	Fecal and blood samples	MASLD compared with HCs: - ↓ α-diversity. - ↓ *Turicibacter*, *Erysipelothrix*, *Parasutterella*, *Acidaminococcus*, *Fastidiosipila*, *Faecalibacterium*, *Biophila*, and *Citrobacter.* - ↓ *Weissella* in obese MASLD only.	N/A
**HUMAN PEDIATRIC STUDIES**				
**Author**	**Population**	**Samples**	**Main Effects on Microbiota**	**Main Effects on Metabolites**
Del Chierico et al., 2017 [67]	61 MASLD (27 MASL, 26 MASH, and 8 obese) and 54 HCs [68]	Fecal and serum samples	- Decreased α-diversity with HCs > obese > MASH > MASL. - ↑ Coriobacteriaceae in MASL. - ↑ *Blautia* in MASH, not MASL or obesity. - ↑ Bacteroidaceae and Bacteroides in obesity. - MASLD compared with HCs: - ↑ *Bradyrhizobium*, *Anaerococcus*, *Peptoniphilus*, *Propionibacterium acnes*, *Dorea*, and *Ruminococcus*. - ↑ Bacteroidetes and Actinobacteria. - ↓ Bacteroidaceae and Bacteroides in MASL/MASH. - ↓ Firmicutes (including *Oscillospira* genus) and Rikenellaceae.	- ↑ metabolites of alcohols, acids, aldehydes, ketones, amines, and esters. - ↓ aromatic hydrocarbons and hydrazines. - ↑ 2-butanone and 4-methyl-2-pentanone in MASH compared with HCs.
Schwimmer et al., 2019 [69]	87 cases (not-MASH, borderline MASH, and definite MASH) and 37 patients with obesity but without MASLD	Fecal and blood samples	- ↔ β-diversity. - ↓ α-diversity in MASH. - ↑ Phyla Bacteroidetes and Proteobacteria. - ↑ Phyla Proteobacteria and TM7 in MASH. - ↑ Genera *Lactobacillus* and *Oribacterium* in MASH. - ↑ Phyla Fusobacteria, Verrucomicrobia, and Lentisphaerae in MASLD. - ↑ Genera *Oscillibacter*, *Lactonifactor*, *Akkermansia*, and *Enterococcus* in MASLD. - ↓ Phylum Firmicutes.	- ↔ Genes in alcohol metabolism. - ↑ Genes for lipopolysaccharide biosynthesis. - ↑ Genes involved in flagellar assembly in moderate-to-severe fibrosis. - ↑ Genes in carbohydrate metabolism in milder disease states.
Yu et al., 2021 [51]	32 MASLD and 36 HCs	Fecal samples	- ↑ Phylum Proteobacteria. - ↑ Species *Escherichia coli*, *Klebsiella pneumoniae*, and *Enterobacter cloacae*. - ↓ Phyla Firmicutes and Bacteroidetes. - ↓ Species *Akkermansia muciniphila*, *Alistipes putredinis*, *Bacteroides uniformis*, *Bacteroides fragilis*, *Oscillibacter* sp. *ER4*, *Ruminococcus bromii*, *Eubacterium ventriosum*, and *Gemmiger formicilis.*	- ↓ bile salt hydrolase (BSH) gene. - ↓ fecal secondary bile acids. - ↑ fecal primary bile acids. - ↑ CDCA-3-β-D-glucuronide and CA/CDCA. - ↓ α-hyodeoxycholic acid, 7-ketolithocholic acid, 23-nordeoxycholic acid, 7,12-diketolithocholic acid (7,12-DiketoLCA), 3-epideoxycholic acid (bDCA), LCA, DHCA, LCA/CDCA, and TLCA/CDCA.

MASLD compared with healthy controls (HCs) unless otherwise stated. NHS, nonalcoholic hepatic steatosis; MASLD, metabolic dysfunction-associated steatotic liver disease; MASH, metabolic dysfunction-associated steatohepatitis; MASL, metabolic dysfunction-associated steatotic liver; CA, cholic acid; BA, bile acid; GCA, glycine-conjugated cholic acid; GCDCA, glycine-conjugated chenodeoxycholic acid; TLCA, taurine-conjugated lithocholic acid; UDCA, ursodeoxycholic acid; CDCA, chenodeoxycholic acid; DCA, deoxycholic acid; LCA, lithocholic acid; DHCA, dehydrocholic acid; ↑, increase; ↓ decrease; N/A, not available.

Disturbances in the gut mycobiome have also been associated with the pathogenesis of MASLD and MASH. A recent study [57] found that patients with MASH had higher levels of *Candida albicans*, *Mucor* sp., *Cyberlindnera jadinii*, *Penicillium* sp., unknown Pleosporales, *Babjeviella inositovora*, and *Candida argentea*, whereas unknown *Saccharomycetales* and *Malassezia* sp. were increased in patients with MASLD. In addition, patients with MASLD and advanced liver fibrosis had significantly higher anti-*C. albicans* IgG levels than controls and patients with F0-F2 fibrosis. Interestingly, in the same study, Western diet-fed GF mice that were colonized with the microbiome from patients with MASH and treated with amphotericin B showed reduced steatohepatitis, inflammation, and fibrosis, supporting the possible role of the mycobiome in MASLD/MASH pathogenesis [57].

Patients with MASLD have been shown to have a lower Firmicutes–Bacteroides ratio (F/B ratio). Elevated levels of Bacteroides are biologically relevant in the pathogenesis of MASLD due to their role in reducing the levels of short-chain fatty acids (SCFAs) and increasing deoxycholic acid, choline, raffinose, and stachyose [70]. Raffinose and stachyose contain fructose, which has been associated with MASLD [71]. Furthermore, high levels of unconjugated bile acids such as deoxycholic acid have been associated with hepatic steatosis [72].

### 4.2. The Intestinal Barrier and the Gut–Liver Axis

The intestinal barrier is a selectively permeable multilayer barrier and is the first point of contact with the intestinal luminal contents. The intestinal barrier comprises the gut microbiome, a mucin-rich mucous layer, an epithelium sealed by tight junctions (claudins, zonula occludens-1 (ZO-1), and occludin), immune cells, and intestinal motility and secretions, as well as the gut–vascular layer and the liver barrier [73,74,75]. The intestinal barrier is tightly interconnected with the liver via the portal vein anatomically and through complex interactions among immune cells, cytokines, and the gut microbiota. This bidirectional connection, known as the gut–liver axis, plays an essential role in modulating the nutrients, substances, and gut-derived antigens that ultimately reach the liver via the portal vein.

Alterations in the integrity of the intestinal barrier have been shown to play an important role in the pathogenesis of MASLD. Individuals with MASLD have increased intestinal permeability, as assessed by serum D-lactate levels, and this correlates with MASLD severity [76]. Factors such as Western diet, alcohol intake, medications, altered bile acid metabolism, and disturbed gut microbiota [77], can disrupt the intestinal tight junctions and the gut–vascular barrier, leading to increased intestinal permeability. This in turn can result in the translocation of bacteria and bacterial metabolites (e.g., lipopolysaccharide [LPS]) to the liver. This endotoxemia stimulates the activation of toll-like receptor 4 (TLR4) [78] and inflammatory signaling cascades including the NF-kB pathway, which results in the production of pro-inflammatory cytokines that promote hepatic inflammation and oxidative stress, which are key components of MASLD pathophysiology [79].

## 5. Metabolic Changes in MASLD

Functional metagenomic analyses have demonstrated metabolite abnormalities in individuals with MASLD [80]. Such metabolites include ethanol, choline, bile acids, and SCFAs, among others. A recent prospective study that followed patients for 4.6 years found differences in the functional profile of the gut microbiota of individuals with MASLD compared with healthy controls, including geranylgeranyl diphosphate biosynthesis, mevalonate pathway, phosphatidate metabolism, and cholic acid degradation [54]. These findings uncover key microbial fingerprints which may be used for the diagnosis of MASLD in early disease stages.

### 5.1. Short-Chain Fatty Acids

Short-chain fatty acids are produced by the colonic gut microbiota through fermentation of undigestible polysaccharides and are generally regarded as beneficial to the host due to their anti-inflammatory effects. They interact with a variety of G-protein-coupled receptors on colonocytes and extra-intestinal organs, including the liver. They serve important roles in energy metabolism, regulating lipid and glucose levels in the blood and affecting immune function [81]. Recent data suggest that SCFAs may also play a role in various diseases including MASLD, including one study [39] in which patients with MASLD were found to have increased levels of acetate, propionate, and butyrate compared with healthy counterparts. These increased levels were associated with lower resting regulatory T cells, as there was a higher Th17/rTreg ratio in peripheral blood.

In another study [82], supplementation with butyrate improved liver steatosis in a population of pre-menopausal females with MASLD. Furthermore, a meta-analysis found that decreased levels of butyrate may contribute to accelerated progression of MASLD [80]. One proposed mechanism through which SCFAs including butyrate may play a role in MASLD is through the inhibition of fatty acid synthesis and the upregulation of beta-oxidation in visceral adipocytes [83]. Other proposed mechanisms focus on the beneficial role of SCFAs in overall gut health, where they serve as nutrition sources for colonocytes [84], stimulate the formation of the mucous layer [85], maintain the integrity of the gut barrier [86], and confer anti-inflammatory effects by inhibiting histone deacetylases. Butyrate in particular has been shown to suppress LPS-induced NF-kB-mediated inflammation [87] and protect against metabolic diseases including hyperinsulinemia, hypertriglyceridemia, and hepatic steatosis [83].

### 5.2. Bile Acids

Bile acids are known for their role in fat emulsification and vitamin absorption in the intestine; however, bile acids also play a role in metabolic functions, such as glucose and lipid modulation. Approximately 85% of primary bile acids are reabsorbed in the ileum and return to the liver via the portal vein (enterohepatic circulation), serving as a negative feedback mechanism to bile acid synthesis. The primary bile acids that ultimately reach the distal part of the ileum and colon are modified, and some are converted into secondary bile acids by the gut microbiota. Secondary bile acids, such as deoxycholic acid (DCA) and lithocholic acid (LCA), are more hydrophobic in nature and are thus more likely to cause noxious effects in the host. They have been shown to play a role in the pathogenesis of MASLD [88]. Compared with healthy controls, individuals with MASLD have increased levels of secondary bile acids and genes involved in secondary bile acid synthesis [59]. Mechanisms through which altered bile acid metabolism can contribute to MASLD development include disruption of the intestinal barrier, as well as activation of Farnesoid X receptor (FXR), Takeda G receptor 5 (TGR5), and sphingosine-1 phosphate receptor 2 (S1PR2) [88].

In a recent study [89], DCA and bile salt hydrolase were increased in the feces of individuals with MASLD, and this was correlated with MASLD severity. DCA has been shown to increase collagen synthesis by hepatic stellate cells [90] and to trigger cell death pathways in the liver, contributing to MASLD development [91]. Murine MASLD models were found to have increased intestinal bile acids [92] and increased serum levels of bile acids, with the latter being correlated with MASLD-associated indices and activated levels of FXR [93], which has been shown to play an important role in MASLD pathogenesis [94]. FXR regulates bile acid homeostasis, as well as hepatic glucose and lipid metabolism [95]. In contrast, decreased levels of secondary bile acids and bile salt hydrolase gene were found in the feces of children with MASLD; however, interestingly, gene abundance for secondary bile acid biosynthesis was significantly higher in children with MASLD [51]. In another study, increased levels of bile acids in the blood were only observed in patients with MASH, and this was related to the degree of insulin resistance [96].

### 5.3. Choline

Choline is a quaternary ammonium alcohol which may be obtained through dietary sources or synthesized endogenously. It is stored and metabolized by the liver into phospholipids, including phosphatidylcholine, which is an essential component of cell membranes. These phospholipids also play important roles in hepatic fat metabolism by solubilizing cholesterol into bile and by facilitating the assembly and excretion of triglycerides in VLDL particles [97]. Deficiencies in choline may result in accumulation of triglycerides in the liver, leading to hepatic steatosis [98]. Animals fed choline-deficient diets have increased oxidative stress, upregulation of inflammatory cytokines, hepatosteatosis, and hepatic fibrosis. This inflammatory state is significantly increased in animals fed diets deficient in both choline and methionine. Specifically, there is increased macrophage infiltrate of the liver and activation of the NF-kB pathway, which stimulates the production of pro-inflammatory molecules including IL-6, TNF, and TGF-B [99]. The reverse has also been demonstrated, where human [100] and animal [99] supplementation with choline appears to reverse hepatic abnormalities.

The gut microbiome metabolizes a myriad of dietary components, including choline. In this manner, it affects the bioavailability of choline and may influence one’s predisposition to developing MASLD. The main bacterial phyla which affect anaerobic choline metabolism in humans include Proteobacteria, Firmicutes, and Actinobacteria [101]. Gut bacteria convert choline to trimethylamine (TMA), which is further oxidized to trimethylamine N-oxide (TMAO). MASLD appears to be characterized by increased conversion of choline into TMA and TMAO, thus resulting in choline deficiency, which, as described above, may influence hepatic steatosis.

### 5.4. Ethanol

MASLD and metabolic dysfunction-associated alcoholic liver disease (metALD) are considered distinct entities despite overlapping clinical presentations. They are distinguished on the basis of alcohol consumption thresholds, typically 30 g or 3 units per day in men and 20 g or 2 units per day in women [102]. Apart from alcohol consumption, the mechanistic explanation for both entities involves lipid accumulation in hepatocytes followed by endoplasmic reticulum stress, mitochondrial dysfunction, cellular apoptosis, inflammation, and fibrosis. Some key hepatic enzymes involved in the pathogenesis of both diseases include SREBP-1c (a transcription factor involved in lipogenesis), carnitine palmitoyltransferase (involved in lipid degradation), and acetyl-CoA carboxylase (involved in de novo lipid generation) [103]. Ethanol impairs liver metabolism by activating SREBP-1c, which stimulates de novo lipid generation via acetyl-CoA carboxylase [103]. Alcohol also inhibits further beta-oxidation through carnitine palmitoyltransferase. This collectively promotes hepatosteatosis [104]. Similar enzymatic activity has been demonstrated in humans with MASLD [105]. Steatosis from de novo lipogenesis in the liver, as well as influx of fatty acids from peripheral tissue (as seen in metabolic syndrome, insulin insensitivity, and obesity), leads to lipotoxicity, which promotes hepatocyte apoptosis and the release of damage associated molecular patterns (DAMPs). These DAMPs release chemokines, cytokines, and other signaling molecules which activate Kupffer cells and stellate cells, recruit inflammatory cells including T cells and neutrophils, and promote macrophage chemotaxis to the liver [106]. Thus, ethanol plays an important role in the pathogenesis of MASLD by upregulating transcription factors and enzymes involved in lipogenesis while suppressing beta-oxidation [107].

The gut microbiome contributes to endogenous ethanol production independently of alcohol consumption through the fermentation of food. In one cohort study, high-alcohol producing *Klebsiella pneumoniae* was implicated in 60% of MASLD subjects. The transplantation of this high-alcohol-producing *Klebsiella pneumoniae* isolate into mice induced MASLD, while the selective elimination of the strain prior to transfer prevented the development of MASLD [108]. In a separate prospective study, the postprandial concentration of ethanol was found to be significantly elevated in the portal vein blood of MASLD patients compared with overweight controls without liver disease after a mixed meal test and an aldehyde dehydrogenase inhibitor. In this same study, Lactobacillaceae was positively correlated with ethanol concentrations [109]. A pediatric study demonstrated overrepresentation of Gammaproteobacteria and *Prevotella* in children with MASLD, both of which have been associated with endogenous alcohol production [110]. Lastly, increased alcohol-producing bacteria have also been implicated in pediatric MASH with overrepresentation of the *Escherischia* genus, which correlated with increased ethanol production [111]. Taken together, these findings suggest that distinct gut microbiome compositions may promote endogenous ethanol production, which may contribute to hepatic lipotoxicity, including the alteration in the activity of enzymes involved in de novo lipogenesis and beta-oxidation, thus promoting steatosis, inflammation, and subsequently fibrosis.

## 6. Treatment

The treatment options for patients with MASLD and MASH have been limited in scope due to the complex and heterogenous pathophysiology associated with the disease. Most common treatments have focused on lifestyle modifications related to physical activity, dietary considerations, and weight loss. Pharmacological treatments have targeted insulin resistance, lipid metabolism, oxidative stress, and hepatic fibrosis [112]. One study [113] showed that MASLD could be induced in germ-free (GF) mice following fecal microbiota transplantation (FMT). In another study [114], GF mice transplanted with the fecal gut microbiota of individuals with MASLD developed increased body weight, higher liver steatosis, and higher cholesterol and triglycerides levels compared with recipients of healthy gut microbiota. These findings have generated considerable interest in the use of microbiome-based treatment options.

### 6.1. Antibiotics

The use of antibiotics in the treatment of MASLD/MASH has been explored in the context of mouse models and human clinical trials. Initial studies demonstrated that the combined administration of polymyxin B and neomycin to fructose-fed mice led to significant decreases in hepatic lipid levels [115]. Furthermore, the use of antibiotic combination treatments involving bacitracin, neomycin, and streptomycin induced significant reductions in liver lipid and triglyceride levels in a high-fat diet (HFD)-induced MASLD mouse model [116]. Recent studies have examined the effect of the non-absorbable antibiotic Rifaximin (commonly used in the treatment of hepatic encephalopathy) with respect to hepatic fibrosis, hepatic lipid accumulation, and intestinal integrity. Choline-deficient/L-amino acid-defined (CDAA) diet-induced MASH mouse models showed ameliorated hepatic fibrosis and improvements in intestinal tight junction integrity following co-administration with Rifaximin and angiotensin receptor blockers (ARBs) [117]. Furthermore, biopsy-identified MASH patients, following treatment with Rifaximin, depicted decreased endotoxins; decreased pro-inflammatory cytokines; decreased liver ALT, AST, GGT, LDL, and ferritin levels; and improved MASLD liver scores [118,119]. The administration of the widely used Gram-negative Triclosan antibiotic also showed improvements in hepatic steatosis, reductions in pathogenic Gram-negative bacteria including Helicobacter pylori and Citrobacter, and an improved Bacteroidetes–Firmicutes ratio in HFD mouse models [120]. Overall, the use of antibiotics has demonstrated efficacy in improving hepatic markers associated with MASLD/MASH; however, the unintended consequences of antibiotic use on intestinal commensal diversity and overall intestinal integrity should be explored.

### 6.2. Probiotics

The use of probiotics and prebiotics in the management and treatment of MASLD and MASH has recently generated considerable interest. Probiotics are non-pathogenic living organisms that may exert positive effects on host health [121]. While numerous probiotic and prebiotic compositions have been explored in the context of MASLD management, most formulations included *Bifidobacterium* and *Lactobacilli* [122]. It has previously been shown by Li et al. that probiotic treatments may improve factors related to liver injury [68]. A common probiotic formulation used in MASLD/MASH is known as VSL#3 and consists of *Bifidobacterium breve*, *Bifidobacterium longum*, *Bifidobacterium infantis*, *Lactobacillus acidophilus*, *Lactobacillus plantarum*, *Lactobacillus paracasei*, *Lactobacillus bulgaricus*, and *Streptococcus thermophiles* [68]. Supplementing HFD feeds with VSL#3 decreased serum ALT levels and improved hepatic insulin resistance in genetically obese mice [68]. A subsequent study used a probiotic/prebiotic formulation consisting of *L. plantarum*, *Lactobacillus delbrueckii* ssp. *bulgaricus*, *L. acidophilus*, *Lactobacillus rhamnosus*, and *Bifidobacterium bifidum* and prebiotics fructose-oligosaccharides on MASH patients for a total of 6 months [123]. This formulation decreased serum ALT levels and increased the Bacteroidetes–Firmicutes abundance ratio [123], which, as noted previously, have implications in the abundance of SCFAs. Overall, probiotics represent a promising treatment option for patients with MASLD/MASH, and further studies addressing different formulations of probiotics and prebiotics will need to be conducted.

### 6.3. Fecal Microbiota Transplantation

Recent studies have explored the possibility of using FMT-based therapies for the amelioration of MASLD. Given that obesity is a significant risk factor for the development of MASLD, differences in gut microbial composition and diversity in lean and obese individuals have been characterized [123,124,125,126]. Lean individuals have an increased prevalence of the Bacteroides phylum, while obese individuals have an increased prevalence of Actinobacteria and Firmicutes [123,124,125,126]. Given that the gut microbiota plays a vital role in metabolism and in the regulation of gut barrier integrity, these differences in bacterial composition have propelled investigations assessing FMT as a potential therapeutic intervention for intestinally involved diseases. In HFD mouse models, it has been shown that 8 weeks of FMT treatment increased cecal butyrate content, increased the presence of ZO-1, alleviated steatohepatitis through decreased hepatic lipid accumulation, and decreased IL-4 and IL-22 pro-inflammatory cytokine levels [127]. Gut microbiota changes in HFD mice were also reversed following FMT treatment with an increased prevalence of beneficial bacterial populations, including *Christensenellaceae* and *Lactobacillus* [127]. Patient studies have also demonstrated that allogeneic FMT can ameliorate intestinal changes associated with MASLD [128]. In one study, MASLD patients received FMT from healthy and thin donors, and while no significant changes were observed with regards to insulin resistance, significant improvements in intestinal integrity as indicated by ZO-1 were noted [128]. Given the significant influence of gut microbial composition and diversity on metabolic and inflammatory pathways, FMT represents a promising therapeutic intervention for the amelioration of microbe-influenced diseases, including MASLD.

## 7. Concluding Remarks

Increasing research is uncovering the alterations in the microbial composition of patients with MASLD and MASH. Alterations in bacterial, fungal, and bacteriophage abundance and diversity affect metabolite composition, including ethanol fermentation, choline and bile acid levels, and SCFA concentrations. These alterations subsequently affect the balance of the innate and adaptive immune systems and promote a pro-inflammatory state, thereby impairing the integrity of the intestinal barrier. Subsequent translocation of the microorganisms and their products across enterocytes and colonocytes results in their introduction to the portal venous blood and ultimately the liver, where hepatic lipid accumulation occurs, resulting in MASLD. Progressive inflammation and hepatic remodeling are mediated by the immune system in response to the hepatosteatosis and drive disease progression to MASH, hepatic fibrosis, and even hepatocellular carcinoma. Emerging research is focusing on the role of microbe-targeting approaches as potential therapeutic avenues for the treatment of MASLD and MASH. Some research is focusing on the use of antibiotics as a means of targeting the overgrowth of specific microorganisms, such as Bacteroidetes and Firmicutes, while other research is focusing on a broader approach of increasing overall microbial diversity. The commonality to these approaches includes manipulating microbial and immune system interactions to achieve a state of homeostasis.

Our understanding of the pathogenesis of MASLD is steadily growing as we unravel the influence of gut microbiota, host metabolism, and environmental factors. Understanding how microorganisms, including bacterial, fungi, and viruses, drive host–microbial dynamics and influence the onset and progression of disease provides clarification on disease pathogenesis while also providing opportunities to develop more targeting treatment paradigms for patients. It opens doors to more treatment approaches in addition to lifestyle interventions. Particular interest is gathering for the use of probiotics, antibiotics, and FMT in altering the intestinal microbiome and restoring host homeostasis. As our understanding of how dysbiosis impacts metabolism and disease continues to grow, future therapies may focus on identifying and targeting this dysbiosis in early stages to prevent and perhaps even reverse MASLD and MASH.
